# Proteomic and phosphoproteomic profiling of COVID-19-associated lung and liver injury: a report based on rhesus macaques

**DOI:** 10.1038/s41392-022-00882-7

**Published:** 2022-01-28

**Authors:** Jiang-Feng Liu, Ya-Nan Zhou, Shuai-Yao Lu, Ye-Hong Yang, Song-Feng Wu, De-Pei Liu, Xiao-Zhong Peng, Jun-Tao Yang

**Affiliations:** 1 State Key Laboratory of Medical Molecular Biology, Department of Biochemistry and Molecular Biology, Institute of Basic Medical Sciences Chinese Academy of Medical Sciences, School of Basic Medicine Peking Union Medical College, Beijing, 100005 China; 2 National Kunming High-level Biosafety Primate Research Center, Institute of Medical Biology, Chinese Academy of Medical Sciences and Peking Union Medical College, Yunnan, China; 3 State Key Laboratory of Medical Molecular Biology, Department of Biochemistry and Molecular Biology, Medical Primate Research Center, Neuroscience Center, Institute of Basic Medical Sciences Chinese Academy of Medical Sciences, School of Basic Medicine Peking Union Medical College, Beijing, China; 4 State Key Laboratory of Proteomics, Beijing Proteome Research Center, National Center for Protein Sciences (Beijing), Research Unit of Proteomics & Research and Development of New Drug of Chinese Academy of Medical Sciences, Institute of Lifeomics, Beijing, 102206 China

**Keywords:** Bioinformatics, Infectious diseases, Infection, Experimental organisms

**Dear Editor**,

The ongoing COVID-19 pandemic, caused by severe acute respiratory syndrome conronavirus 2 (SARS-CoV-2), has led to over 209,201,939 confirmed cases and 4,390,467 deaths all over the world as of 19 August 2021 (https://covid19.who.int/). Novel therapeutic agents and vaccines are desperately needed and mechanism exploration is imperative. Though clinical tissues are preferred samples for molecular and mechanism study, COVID-19 clinical tissues are rare and mostly come from autopsies of end-stage patients.^1,2^ Animal models, especially nonhuman primate models, are therefore constructed for SARS-CoV-2-associated research.^3^

Proteomic analysis has proven to be an effective technology to comprehensively understand COVID-19 induced organic response^1,2^ but has not been widely applied to study animal models affected with SARS-CoV-2. In this study, we performed the first proteomic and phosphoproteomic analysis for rhesus monkeys infected with SARS-CoV-2 to glance at COVID-19-associated molecular mechanisms in nonhuman primates. We focused on lung and liver because the lung is the major organ affected by SARS-CoV-2 and almost 50% of COVID-19 patients have symptoms of liver injury.^4^

Eight rhesus monkeys (Supplementary Table S1) were divided into blank control and virus-infected group. On 7 days post-infection (dpi), animals were euthanized and their tissues were harvested for viral load detection, morphological analyses (Supplementary Fig. S1a-e), and proteomic and phosphoproteomic analyses (Fig. 1a).

**Fig. 1:**
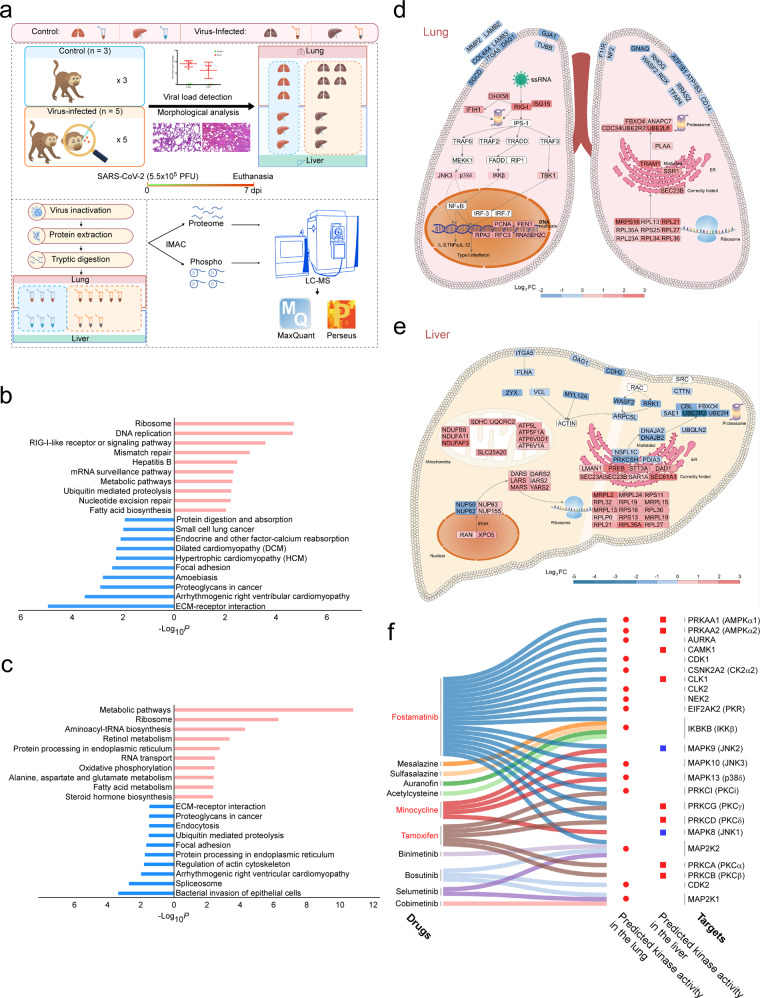
Proteomic, phosphoproteomic, and bioinformatic analyses of rhesus macaques with COVID-19. **a** Brief workflow of this study. **b** KEGG enrichment analysis of differentially expressed proteins in the lung of control and virus-infected rhesus macaques. Red: upregulated. Blue: downregulated. **c** KEGG enrichment analysis of differentially expressed proteins in the liver of control and virus-infected rhesus macaques. Red: upregulated. Blue: downregulated. **d**, **e** Dominant pathways in the lung (**d**) and liver (**e**). Red boxes with black border: upregulated proteins in the infected tissue. Blue boxes with black border: downregulated proteins in the infected tissue. Pink boxes with pink border: kinases predicted to be activated in the infected tissue. White boxes: proteins in the pathway but not in the differentially expressed proteins. **f** Correspondence between the predicted kinases and FDA-approved drugs in DrugBank. Red marked drugs were predicted to work in both the lung and the liver. Red circles represent predicted activated kinases in the lung. Red and blue squares represent predicted activated and inhibited kinases in the liver, respectively

Proteomics quantified 6715 proteins in the lung and 5238 ones in the liver (Supplementary Tables S2–S4 and Supplementary Fig. S2a). Principle component analysis (PCA) showed that the overall difference of protein expression pattern between control and virus-infected groups in the liver was larger than that in the lung (Supplementary Fig. S2b, c). Compared to the control, 757 proteins in the lung (supplementary Table S5 and Supplementary Fig. S2d) and 1219 ones in the liver (Supplementary Table S6 and Supplementary Fig. S2e) were differentially expressed in the infected group (Supplementary Table S7 and Supplementary Figs. S2f–S3). KEGG enrichment analysis were performed for differentially expressed proteins. In the infected lung, we observed that the upregulated proteins were enriched for ribosome, DNA replication, RIG-I-like receptor or signaling pathway, mismatch repair, metabolic pathways, etc.; the downregulated proteins were most closely related to ECM-receptor interaction (Supplementary Table S8 and Fig. 1b). As for the liver, the upregulated proteins accompanied by SARS-CoV-2 infection were enriched for multiple metabolic pathways, ribosome, aminoacyl-tRNA biosynthesis, and oxidative phosphorylation, etc.; the downregulated proteins were related to spliceosome, regulation of actin cytoskeleton, and focal adhesion, etc. (Supplementary Table S9 and Fig. 1c).

Proteins in the enriched pathways were submitted to STRING database for protein-protein interaction (PPI) analysis. Both the lung (Supplementary Fig. S4a) and the liver (Supplementary Fig. S4b) had elevated levels of metabolism-related proteins and closely connected ribosomal proteins after infection. Combining KEGG and STRING database, we saw that upregulated proteins presented quite a few details of RIG-I pathway, protein processing, and DNA replication and repair in the infected lung (Fig. 1d). Upregulated RIG-I, ISG15, DHX58, IFIH1, and TBK1 accounted for activation of RIG-I pathway, which is a typical virus infection-induced pathway. Together with ribosomal proteins, proteins in ER membrane (SEC23B, SSR1 and TRAM1) and components of proteasome (FBXO4, ANAPC7, CDC34, UBE2R2, and UBE2L6) could participate in protein synthesis, transport, and degradation. PCNA, RPA2, RFC3, FEN1, and RNASEH2C mainly locate in the nucleus and their upregulation contributed to potentially enhanced DNA replication and repair in the lung during SARS-CoV-2 infection (Fig. 1d).

Alterations of RIG-I pathway members or DNA replication and repair factors were not significant in the liver (Fig. 1e), though high viral loads were also detected in this organ; nevertheless, we found a number of differentially expressed proteins in the oxidative respiratory chain and protein processing. Components of the ATP synthase and three oxidative respiratory chain complexes were identified. Robust elevation (FC > 2.0) of NDUFB8, NDUFA11, and NDUFAF3 in complex II, SDHC in complex III, UQCRC1 and UQCRC2 in complex IV, and ATP5L, ATP5FA1, and ATP6V0D1 in the ATP synthase prompted that the infected liver experienced enhanced oxidative phosphorylation process. XPO5 and RAN, which were required for translocation of RNA and proteins through the nuclear pore complex, and significantly differentially expressed amino acid-tRNA ligases (e.g., DARS, DARS2, LARS, IARS2, MARS, and YARS2) were all upregulated. Together with the upregulated ribosomal proteins, it suggested enhanced protein synthesis happened in infected livers (Fig. 1e). Proteins assisting polypeptides in entering or budding from ER, such as PREB, STT3A, SEC23A, SEC23B, SAR1A, and SEC61A1 were elevated in infected livers, indicating a smooth transport process of correctly folded protein in ER. However, misfolded protein processing-associated proteins (NSFL1C, DNAJA2, DNAJB2, and UBQLN2) and components of proteasome (CBL, FBXO4, SAE1, UBE2R2, and UBE2H) were downregulated in the liver. It seemed that protease-mediated protein degradation was active in the lung but inhibited in the liver.

Proteins around focal adhesions tended to decrease on 7 dpi after SARS-CoV-2 infection. ITGA5 which belongs to the integrin alpha family and DAG1 which links ECM and cytoskeleton decreased in both the infected lung and liver. However, among the downregulated proteins, the lung contained more ECM (COL4A4, LAMB2, LAMB3, and MMP2) and cellular membrane ones (GJA1, ATP1B1, and ATP1B3; Fig. 1d) while the liver contained more actin-regulated ones (MYL12A, WASF2, BRK1, and ARPC5L; Fig. 1e).

Phosphorylation is a quick regulatory method for cells to response to stimulation and has been detected in some SARS-CoV-2-infected cell models or autopsies. We therefore analyzed phosphoproteome (Fig. 1a) to supplement the molecular network and find potential drug targets during SARS-CoV-2 infection. We quantified 12,418 phosphosites in 4185 proteins in the lung and 8134 sites in 3191 proteins in the liver (Supplementary Tables S10–S12 and Supplementary Fig. S5a). PCA showed that the overall phosphorylation patterns were slightly different in control and virus-infected organs (Supplementary Figs. S5b–S5c). We found 1162 sites in 873 proteins in the lung (Supplementary Table S13 and Supplementary Fig. S5d) and 960 sites in 712 proteins in the liver (Supplementary Table S14 and Supplementary Fig. S5e) were differentially expressed (Supplementary Table S15 and Supplementary Figs. S5f–S6). In the lung, proteins with upregulated phosphosites were enriched for metabolic pathways, spliceosome, and ubiquitin-mediated proteolysis while proteins with downregulated sites were associated with Fc gamma R-mediated phagocytosis, adherent junction, and basal transcription factors (Supplementary Table S16 and Supplementary Fig. S7a). As for the liver, proteins with upregulated phosphosites were enriched for multiple metabolic pathways while proteins with downregulated sites were related to basal transcription factors, spliceosome, and RNA transport, et al. (Supplementary Table S17 and Supplementary Fig. S7b). Proteins in the enriched KEGG pathways were submitted to STRING for PPI analysis. We saw more upregulated phosphosites than downregulated ones existed among the interacted proteins, but there was no clear consistence trend between the FC of protein in the proteome and that of phosphosite in the phosphoproteome (Supplementary Fig. S7c, S7d). We specifically extracted protein/phosphosite pairs which had opposite FC in the proteome and phosphoproteome (Supplementary Fig. S8a, S8b). We also screened cathepsins, TMPRSS2, and S100 calcium-binding proteins, which were reported to play key roles in the entrance of SARS-CoV-2 into cells, in each omics and found most of these molecules did not change significantly on 7 dpi in macaques. The infected lung had significantly upregulated S100P and the liver had upregulated CTSD. S100A9 were significantly downregulated in both the lung and the liver (Supplementary Fig. S8c, d). ACE2 was detected in none of these omics.

To obtain extended information, we performed kinase prediction using all the identified phosphosites in each organ with the help of NetworKIN. In the lung, we found that kinases related to inflammation (PKR, IKKβ, P38δ, and JNK3; Fig. 1d), cell cycle (CDK1 and CDK2), proliferation (MAP2K1 and MAP2K2), and metabolism (AKT3, PKCi, and AMPKs) were activated (Supplementary Table S18 and Supplementary Fig. S9a) while cytoskeletal reorganization-associated kinase, MRCKα, was inhibited. As for the liver, we saw metabolism-related kinases (AMPKs and PKCs) were activated but inflammation-related ones were quiescent or even inhibited (JNK1 and JNK2; Supplementary Table S19 and Supplementary Fig. S9b). Not all predicted kinases could be found in the original proteome and phosphoproteome data (Supplementary Fig. S9c, S9d), suggesting kinase prediction analysis might expand targets for SARS-CoV-2 exploration. We focused on compounds in DrugBank and found 11 FDA-approved drugs might work on the predicted kinases (Supplementary Table S20 and Fig. 1f). Fostamatinib, minocycline, and tamoxifen were predicted in both the lung and the liver. Fostamatinib, bosutinib, and N-acetylcysteine have been tested in COVID-19-associated cell models or clinical trials in recent work.^5^

We also compared this research with some previous reports (Supplementary Tables S21–S25 and Supplementary Fig. S10). We think COVID-19-associated work from different research teams could complement each other.

In conclusion, this study provided the first proteomic and phosphoproteomic profiling of SARS-CoV-2-infected lung and liver tissues in rhesus macaque. Compared to the autopsy samples of human beings, rhesus macaque models offered us a chance to investigate molecular alterations in tissues at early stage of infection. We observed potentially active protein synthesis and processing, and dysregulated ECM, cell-to-cell junction, and cytoskeleton but no fibrosis in both the infected lung and liver. Relatively, the lung experienced strong inflammatory response while the liver had enhanced oxidative phosphorylation and metabolic processes during infection. Though limited annotation of proteins in rhesus macaques and no more validation experiments led to some limitations, this study offered valid data resource for further mechanism and drug discovery research for SARS-CoV-2.

## Supplementary information

Supplementary_materials

Table S1

Table S2

Table S3

Table S4

Table S5

Table S6

Table S7

Table S8

Table S9

Table S10

Table S11

Table S12

Table S13

Table S14

Table S15

Table S16

Table S17

Table S18

Table S19

Table S20

Table S21

Table S22

Table S23

Table S24

Table S25

## Data Availability

All proteomics and phosphoproteomics raw data have been deposited to the ProteomeXchange Consortium with the identifier PXD027179 and PXD027180. All data supporting the findings of this study are available from the corresponding author on reasonable request.
