# Supplementary material for: Proteomic and phosphoproteomic profiling of COVID-19-associated lung and liver injury: a report based on rhesus macaques
Source: Signal Transduct Target Ther. 2022 Jan 28;7:27. doi: 10.1038/s41392-022-00882-7 (PMC8795284; doi:10.1038/s41392-022-00882-7)
Supplement: Supplementary file 1 — Supplementary_materials [file 41392_2022_882_MOESM1_ESM.docx]

Supplementary Materials for

Proteomic and phosphoproteomic profiling of COVID-19-associated lung and liver injury: a report based on rhesus macaques

Jiang-Feng Liu^1^, Ya-Nan Zhou^2,3^, Shuai-Yao Lu^2,3*^, Ye-Hong Yang^1^, Song-Feng Wu^4^, De-Pei Liu^1*^, Xiao-Zhong Peng^2,3*^, Jun-Tao Yang^1*^

Correspondence to: Shuai-Yao Lu (lushuaiyao-km@163.com), De-Pei Liu (liudp@pumc.edu.cn), Xiao-Zhong Peng (pengxiaozhong@pumc.edu.cn), or Jun-Tao Yang (yangjt@pumc.edu.cn)

Jiang-Feng Liu, Ya-Nan Zhou, and Shuai-Yao Lu contributed equally to this work.

^*^Shuai-Yao Lu, De-Pei Liu, Xiao-Zhong Peng, and Jun-Tao Yang are corresponding authors to this work.

**This PDF file includes:**

Supplementary Materials and Methods

Supplementary Figures S1 to S10

**Other Supplementary Materials for this manuscript include the following:**

Supplementary Table S1 to S25

**Materials and methods**

Ethics and biosafety statement

All animal procedures in this article were approved by the Institutional Animal Care and Use Committee of Institute of Medical Biology, Chinese Academy of Medical Science (ethics number: DWSP202002 001). All animal performed in accordance with the guidelines for the National Care and Use of Animals approved by the National Animal Research Authority and the ABSL-4 facility of the National Kunming High-level Biosafety Primate Research Center, Yunnan, China.

Virus amplification and identification

SARS-CoV-2 was derived from the Center of Disease Control and Prevention of Guangdong Province, one of the strains named as “GD108#”. Viruses were amplified in Vero E6 cells, purified, and concentrated with an ultrafilter system with a 300-kDa module (Millipore, US). SARS-CoV-2 was confirmed via reverse-transcription polymerase chain reaction (RT-PCR), sequencing, and transmission electronic microscopy, titrated via a plaque assay (10^7^ plaque-forming units [PFU]/mL).

Animal experimental procedures

Eight rhesus monkeys were used in this study and animal information is detailed in supplementary Table S1. Animals were divided into two groups: the virus infection group intranasal inoculation with SARS-CoV-2 (Monkey ID, HHH-1/2/3/4/5) and the blank control group without treatments (Monkey ID, HHH-6/7/8). Before viral inoculation, the animals were anesthetized with ketamine (6mg/kg). Each animal in the virus infection group was challenged with 1 mL of 5.5 × 10^5^ PFU SARS-CoV-2 (500 μL/each nostril). Animals were dissected on 7 dpi, and tissue samples were harvested for viral loads, histopathology, and proteomic and phosphoproteomic analyses.

Morphological analysis

Chest X-ray image of each anesthetized animal was taken at 55–75 V and 8–12.5 mA using a MobileCooper mobile digital medical X-ray photography system (Browiner, China). X-ray image were independently evaluated in a double-blind manner by two radiologists. Photos of liver were taken with a digital camera. The liver surface was cleaned, and then placed in a well-lit place.

Tissue samples of lung and liver were harvested and fixed in 10% neutral-buffered formalin for 3-7 days. Formalin-fixed paraffin-embedded tissues were cut into 5 μm sections for H & E staining and histopathologic analysis. The histopathological changes of lung were observed under microscope by two pathologists with double blindness.

Viral RNA extraction and quantification of the viral RNA genome

Trizol suspension of 400 μL swab samples or 100 mg tissue samples from each animal was used for RNA extraction using a Direct-zol RNA Miniprep Extraction Kit (Zymo Research, catalog No. R2052) according to the manufacturer’s instructions. Swab samples soaked in Trizol solution were vortexed and then swabs were removed. The Trizol suspension of swab samples (400 μL) were used for RNA extraction, which was washed with 50 μL DNase/RNase-free water to elute RNA and stored at -80 ℃. For tissue homogenization, 1 mL Trizol were added to 100 mg tissue. The supernatant (400 μL) was centrifuged to extract RNA template, which was washed with 50 μL DNase/RNase-free water to elute RNA and stored at -80 ℃. Real-time RT-PCR was used to quantify the viral genome using TaqMan Fast Virus 1-Step Master Mix (Thermo Fisher Scientific), and SARS-CoV-2 RNA was used as a standard curve. Real-time RT-PCR was performed on a CFX384 Touch Real-Time PCR Detection System (Bio-Rad). Primers and probe sequences were derived from N gene (forward: 5’-GGGGAACTTCTCCTGCTAGAAT-3’, reverse: 5’-CAGACATTTTGCTCTCAAGCTG-3’, probe: 5’-FAMTTGCTGCTGCTTGACAGAT-TAMRA-3’) according to the sequence recommended by WHO and China CDC. RT-PCR was conducted under the following conditions: 25 ℃ for 120 s, 50 ℃ for 15 min, 95 ℃ for 120 s, and 40 cycles at 95 ℃ for 5 s and 60 ℃ for 30 s.

Sample preparation and LC-MS/MS analysis

About 150 mg tissue sample from each animal was used for protein extraction. The tissue samples were added with 4 times volume of lysis buffer (1% SDS, 1% protease inhibitor, and 1% phosphatase inhibitor), homogenized, and heated for virus inactivation. After ultrasonic lysis, the remaining debris was removed by centrifugation at 12,000 × *g* at 4 ℃ for 10 min. The supernatant was collected and the protein concentration was determined with a BCA kit.

For digestion, the protein solution was reduced with 5 mM dithiothreitol for 30 min at 56 ℃ and alkylated with 11 mM iodoacetamide for 15 min at room temperature in darkness. Then, the urea concentration of the sample was diluted to less than 2 M. After that, the trypsin was added at 1:50 trypsin-to-protein mass ratio for the first digestion overnight and 1:100 trypsin-to-protein mass ratio for a second 4 h-digestion. Finally, the peptides were desalted and pending for subsequent peptide fractionation.

The peptide sample for proteome analysis were fractionated into fractions by high pH reverse-phase HPLC using Agilent 300 Extend C18 column (5 μm particles, 4.6 mm ID, 250 mm length). Briefly, peptides were firstly separated with a gradient of 8% to 32% acetonitrile in 10 mM ammonium bicarbonate pH 9 over 60 min into 60 fractions. Then, the peptides were combined into 10 fractions and dried by vacuum centrifuging.

For phosphopeptide enrichment, peptide mixtures were firstly incubated with Fe-IMAC microspheres suspension with vibration in loading buffer (50% acetonitrile/6% trifluoroacetic acid). The Fe-IMAC microspheres with enriched phosphopeptides were collected by centrifugation. To remove nonspecifically absorbed peptides, the Fe-IMAC microspheres were washed with 50% acetonitrile/6% trifluoroacetic acid and 30% acetonitrile/0.1% trifluoroacetic acid sequentially. To elute the enriched phosphopeptides, elution buffer containing 10% NH_4_OH was added and the enriched phosphopeptides were eluted with vibration. The supernatant containing phosphopeptides was collected and lyophilized for LC-MS/MS analysis.

An Orbitrap Exploris^TM^ 480 mass spectrometer (Thermo Fisher Scientific) equipped with EASY-nLC 1200 UPLC system was used for tandem mass spectrometry (MS/MS) analysis. A binary buffer system, consisting of buffer A (0.1% formic acid in water) and buffer B (0.1% formic acid in 90% acetonitrile) was used for peptide separation. The FAIMS device was placed between the nanoelectrospray source and the mass spectrometer. The electrospray voltage was applied at 2.3 kV. The intact peptides were detected in the orbitrap at a resolution of 60,000. Peptides were then selected for MS/MS with NCE 27 and the fragments were detected in the orbitrap at a resolution of 30,000. A data-dependent procedure that alternated between one MS scan followed by 25 MS/MS scans with 20 s dynamic exclusion. For phosphoproteome analysis, the automatic gain control (AGC) was set at 100%, with an intensity threshold of 2E4 and a maximum injection time of 50 ms. For proteome analysis, the intensity threshold as set as 5E4.

Database search and bioinformatics analysis

The resulting MS/MS raw files were processed using MaxQuant search engine (v1.6.5.0) ^1^ against Macaca mulatta database (Uniprot, 20201216 released) concatenated with reverse decoy database. Trypsin/P was specified as cleavage enzyme allowing up to 2 missing cleavages. Carbamidomethyl (C) was specified as fixed modification. Oxidation (M), acetylation (protein N-terminal), deamidation (NQ), and phosphorylation (STY) were specified as variable modifications. FDR was adjusted to < 1% and minimum score for modified peptides was set > 40. Minimum peptide length was set at 7. For quantification method, match between run was enabled. All the other parameters in MaxQuant were set to default values.

MaxQuant derived data were submitted to Perseus ^2^ for subsequent processing. First, proteins matched to potential contaminant and reverse database were excluded. Then, only phosphosites whose localization probabilities > 0.75 were kept. Proteins/phosphosites which existed in > 50% samples in control or virus-infected group were considered as quantifiable. Normalization and imputation were performed using “Divide” (by median of column) and “Replace missing values from normal distribution” functions in Perseus. The obtained matrix was used for differential expression analyses.

Student’s *t* test was used to find out the differentially expressed proteins/phosphosites and the cutoff was set at *p* < 0.05. Fold change (FC) was calculated by dividing the mean values of virus-infected group by that of control group (FC = mean of virus-infected group/mean of control group). Proteins/phosphosites with *p* < 0.05 and FC > 1 were considered as upregulated, and those with *p* < 0.05 and FC < 1 were downregulated.

Upregulated and downregulated proteins were submitted to KOBAS (http://kobas.cbi.pku.edu.cn/) ^3^ for KEGG ^4^ enrichment analysis (statistical method = hypergeometric test/Fisher’s exact test; FDR correction method = Benjamini and Hochberg). Pathways with *p* value < 0.05 were set as enriched ones (supplementary Table S6 and S7). The proteins in enriched pathways were also submitted to STRING database (https://string-db.org/) for protein-protein interaction analysis to identify potential protein complexes or clusters.

Kinase prediction was performed using NetworKIN ^5,6^ following the algorithm’s instructions based on all identified phosphosites in the lung or liver. Based on the Gene Set Enrichment Analysis (GSEA) principle,^7^ kinase activity was calculated and matched to the human kinome tree. Kinome tree modified courtesy of Cell Signalling Technology Inc. (www.cellsignal.com) and annotated using Kinome Render.^8^

Drug targets with FDA-approved drugs were extracted from the DrugBank database (https://go.drugbank.com/, released on 2021.01.03).

**Supplementary figures**


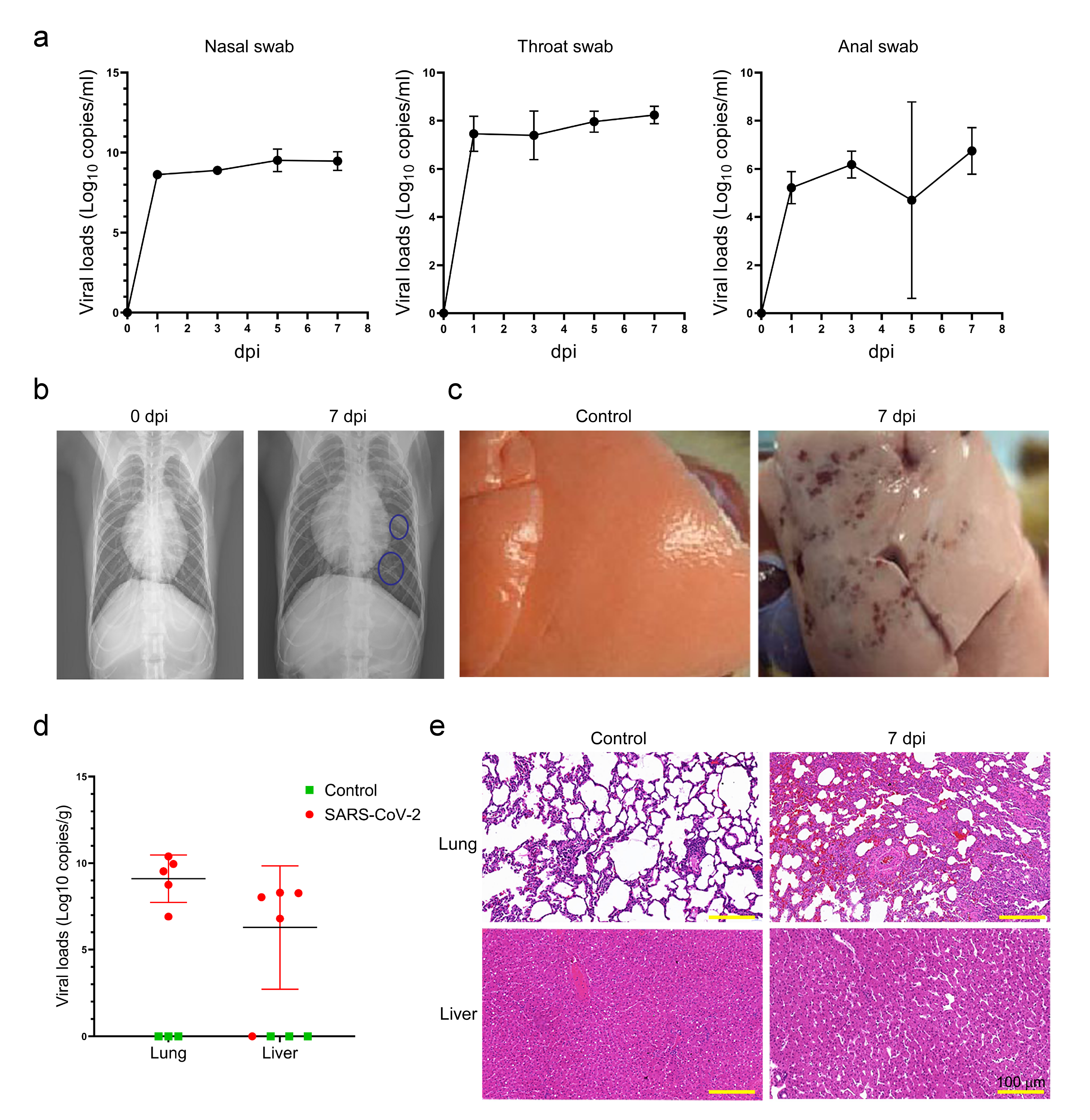


**Supplementary Figure. S1** Verification of SARS-CoV-2 infection and subsequent lesions in the lung and liver of rhesus macaques.

a. SARS-CoV-2 RNA was detected in nasal, throat, and anal swabs by quantitative RT-PCR for the five virus-infected animals.

b. Representative chest x-rays of rhesus macaques in the blank control group and the virus-infected group. Areas of interstitial filtration and exudative lesion are highlighted with blue circles.

c. Representative digital images of lungs harvested from the blank control and the virus-infected animals. Compared to the control, the infected lungs had multiple pathological lesions and bleeding points.

d. SARS-CoV-2 RNA was detected by quantitative RT-PCR in the lung and liver homogenate. All the five lungs from the infection group had virus detected, but only four livers had virus and the viral loads in livers were lower than that of lungs.

e. Representative H & E staining images for control and SARS-CoV-2 infected lung and liver tissues in rhesus macaques (scale bar = 100 μm). The infected lungs had apparent inflammatory cells infiltration, local thickening of pulmonary septum, and thrombosis in the vessels compared to the control ones. In the infected livers, we observed widening of hepatic sinusoidal space and scattered infiltration of inflammatory cells.


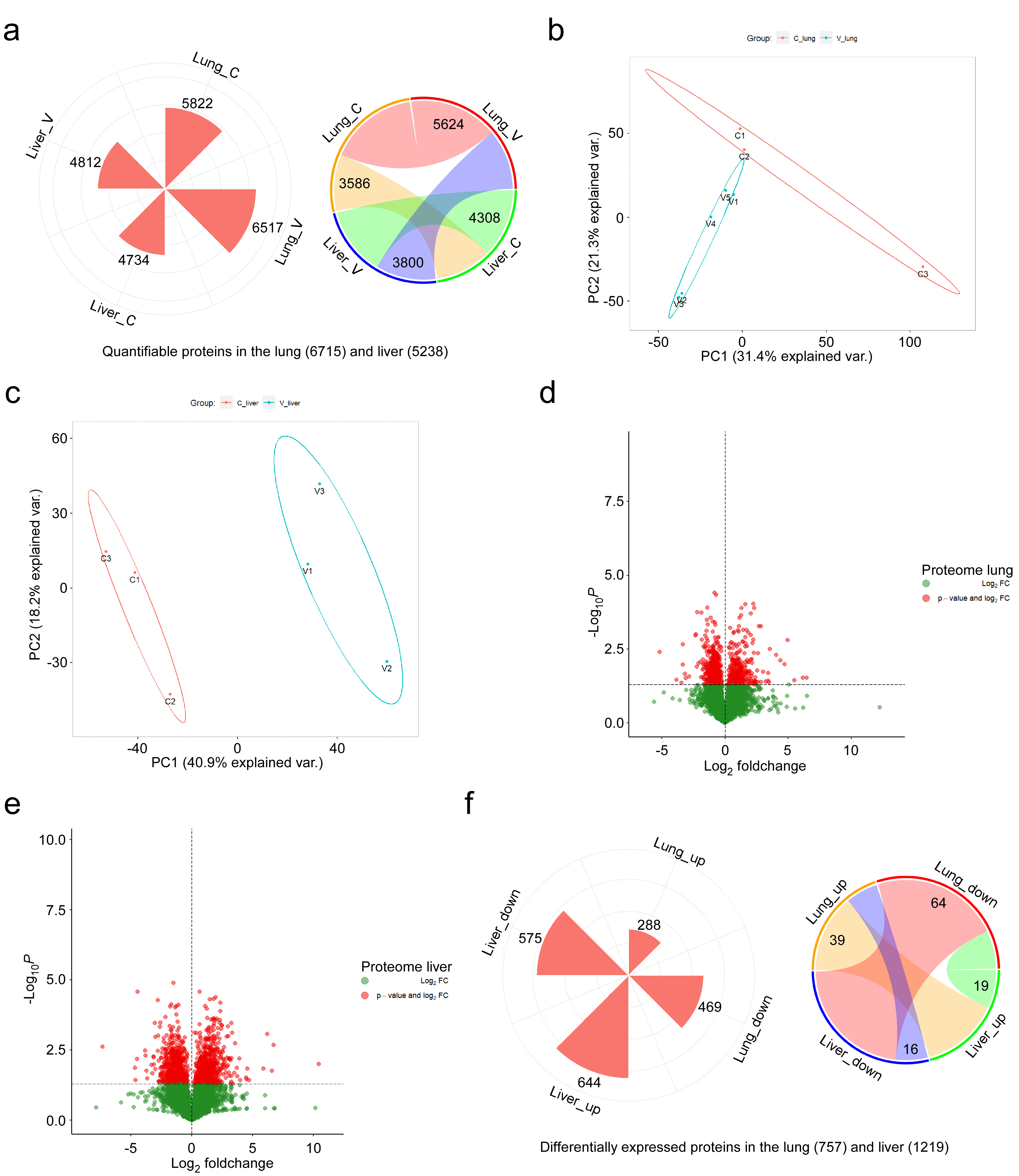


**Supplementary Figure. S2** Proteomics profiling of lung and liver tissues from SARS-CoV-2-infected and control rhesus macaques.

a. Quantified proteins in the lung and liver. The left panel shows the counts of quantifiable proteins in each group, including the control lung and liver and the virus-infected lung and liver. The right panel shows the intersection of quantifiable proteins between each group. Totally, 6,715 proteins were quantified in the lung (control: 5,822; virus-infected: 6,517; see also supplementary TableS2) and 5,238 ones were in the liver (control: 4,734; virus-infected: 4,812; see also supplementary TableS3).

b. PCA analysis of the pulmonary proteome profile. Each point represents one lung tissue sample.

c. PCA analysis of the hepatic proteome profile. Each point represents one liver tissue sample.

d. Pairwise comparison of each protein between control and virus-infected groups was performed with Student’s *t* test. The *p* value and fold change of each quantified protein in the lung was plotted. The cutoff of differentially expressed proteins was set as *p* value < 0.05.

e. Pairwise comparison of each protein between control and virus-infected groups was performed with Student’s *t* test. The *p* value and fold change of each quantified protein in the liver was plotted. The cutoff of differentially expressed proteins was set as *p* value < 0.05.

f. Differentially expressed proteins in the lung and liver. The left panel shows the counts of differentially expressed proteins in the lung and liver. We divided mean values of virus-infected group by mean values of control group to get fold change (FC). Up is set as *p* value < 0.05 and FC > 1 while down is set as *p* value < 0.05 and FC < 1. The right panel shows the intersection of differentially expressed proteins between each dataset. Compared to the control, 757 proteins in the lung (288 upregulated, 469 downregulated; see also supplementary TableS4) and 1,219 ones in the liver (644 upregulated, 575 downregulated; see also supplementary TableS5) were differentially expressed in the infected group.


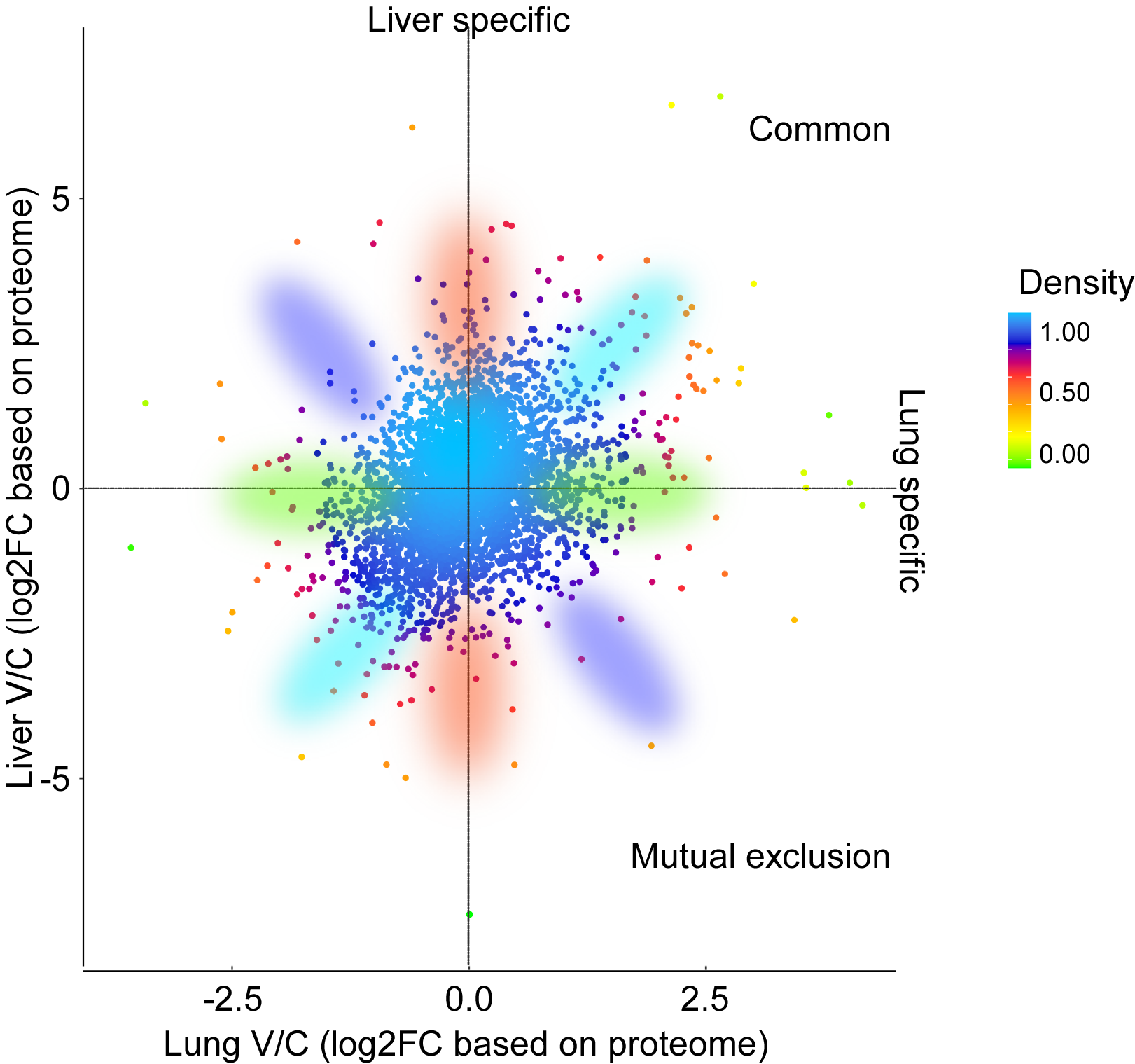


**Supplementary Figure. S3** Comparison between the shared proteins in the lung and liver of rhesus macaques after SARA-CoV-2 infection.

The intersection of quantifiable proteins in the lung and liver were extracted and their fold changes in these two organs after infection were used to construct the scatter plot. The green marked parts represent proteins that were differentially expressed only in the lung (lung specific). The orange marked parts represents proteins that were differentially expressed only in the liver (liver specific). The blue and purple marked parts represent proteins that were differentially expressed both in the lung and liver. Blue marks mean the fold changes of proteins have coincident trends in both the lung and liver after SARS-CoV-2 infection (common). Purple marks mean the fold changes of proteins have opposite trends in the lung and liver after infection (mutual exclusion).


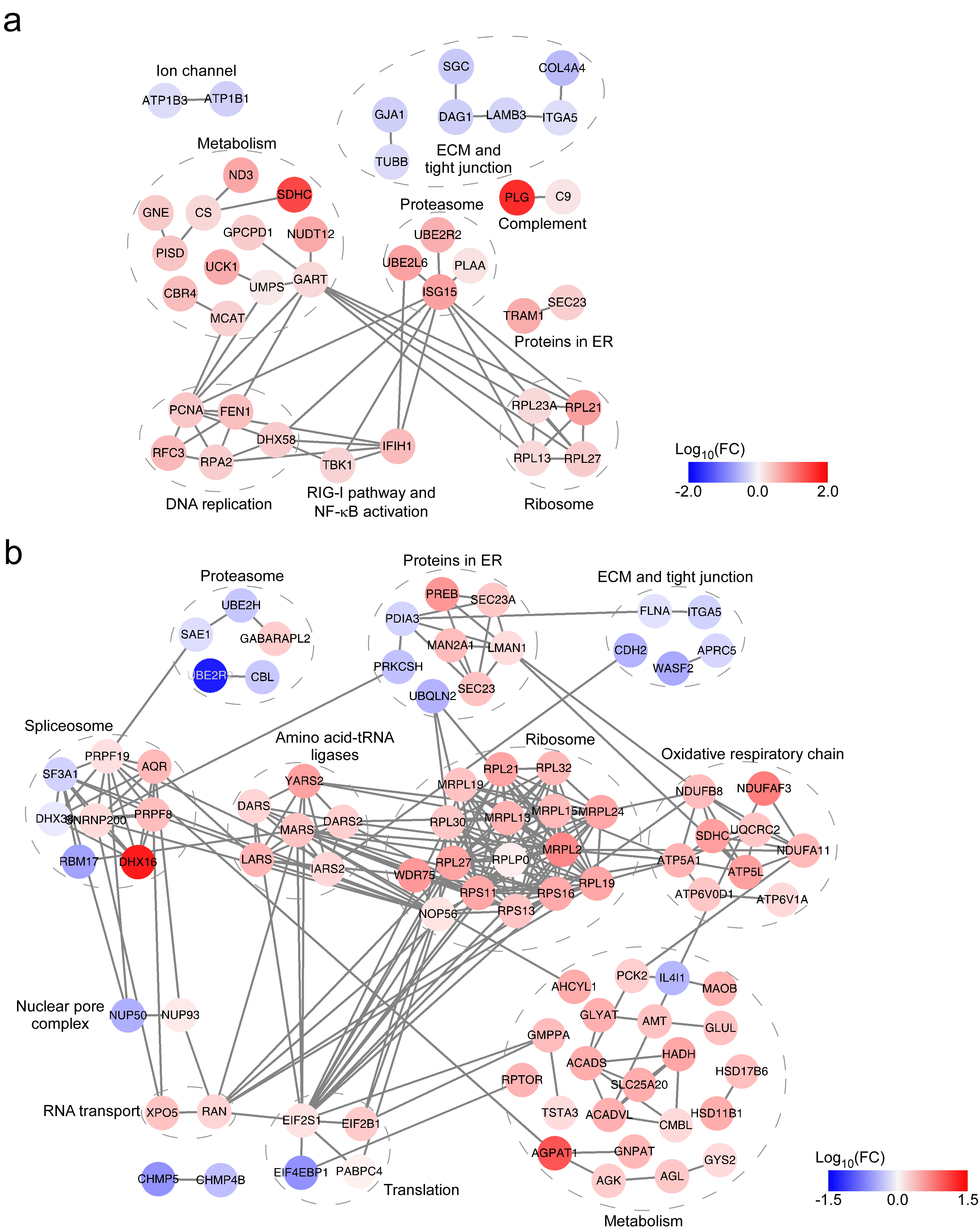


**Supplementary Figure. S4** PPIs among proteins in the enriched KEGG pathways.

The color represents the relative value of log_10_(FC). Red means the protein was upregulated in the infected tissues, and blue means the protein was downregulated.

a. PPIs among proteins in the enriched pathways in the lung of rhesus macaques. Upregulated proteins were mainly associated with DNA replication, RIG-I and NF-κB pathways, protein progression from ribosome and ER to proteasome, and metabolism. Downregulated proteins were clustered into ECM, tight junction, and ion channel, which mainly locates around the cellular membrane.

b. PPIs among proteins in the enriched pathways in the liver of rhesus macaques. Upregulated proteins were mainly clustered into oxidative respiratory chain, metabolism, ribosome, and amino acid-tRNA ligases. Downregulated proteins were mainly associated with proteasome, ECM, and tight junction. Dysregulated proteins involved in protein and RNA processing, including nuclear pore complex, spliceosome, and ER, were also observed.


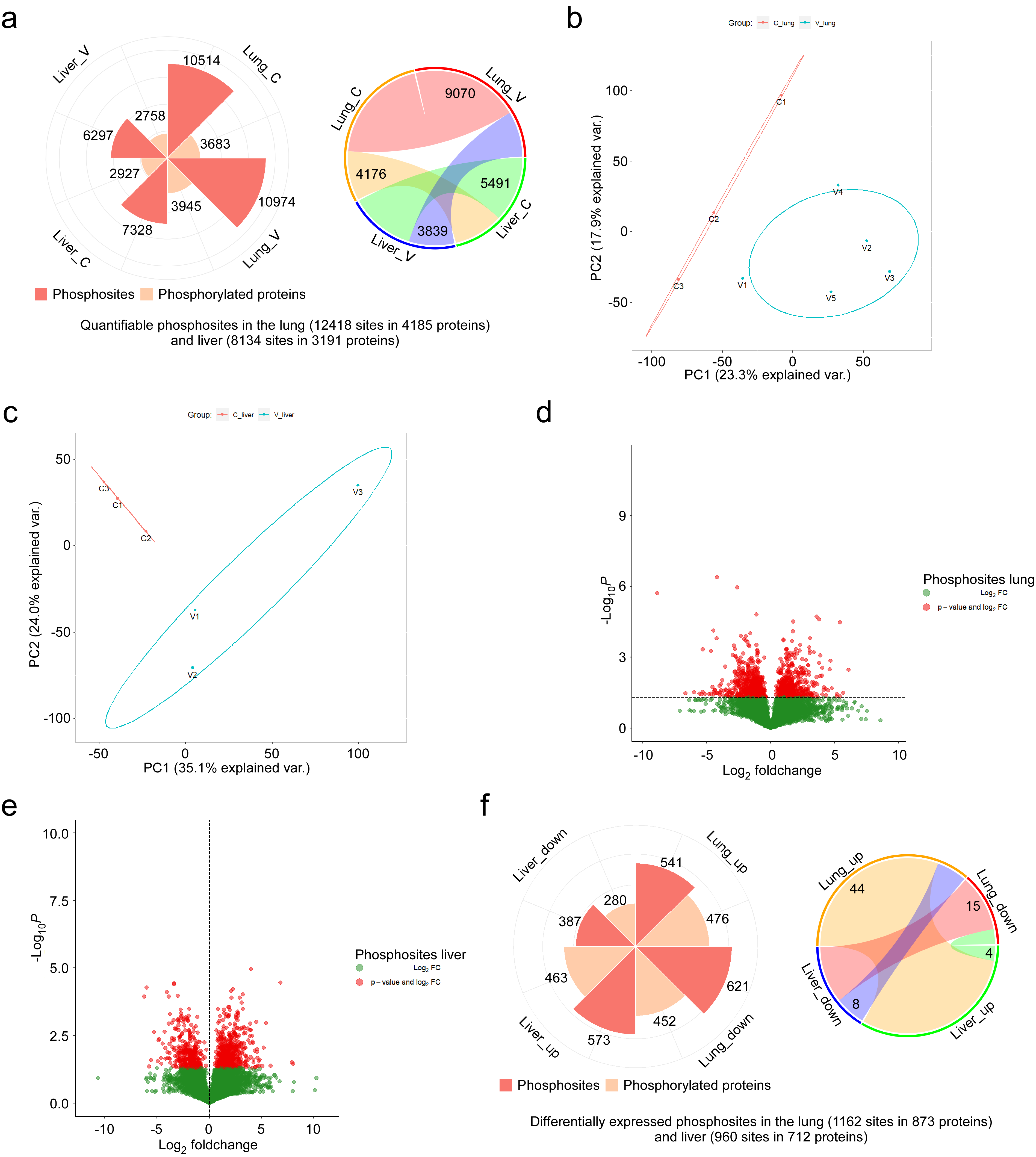


**Supplementary Figure. S5** Phosphoproteomics profiling of lung and liver tissues from SARS-CoV-2-infected and control rhesus macaques.

a. Quantified phosphosites in the lung and liver. The left panel shows the counts of quantifiable phosphosites in each group, including the control lung and liver and the virus-infected lung and liver. The right panel shows the intersection of quantifiable phosphosites between each group. Totally, 12,418 phosphosites (control: 10,514; virus-infected: 10,974; see also supplementary TableS8) in 4,185 proteins in the lung and 8,134 sites (control: 7,328; virus-infected: 6,297; see also supplementary TableS9) in 3,191 proteins in the liver were quantified.

b. PCA analysis of the pulmonary phosphoproteome profile. Each point represents one lung tissue sample.

c. PCA analysis of the hepatic phosphoproteome profile. Each point represents one liver tissue sample.

d. Pairwise comparison of each phosphosite between control and virus-infected groups was performed with Student’s *t* test. The *p* value and fold change of each quantified site in the lung was plotted. The cutoff of differentially expressed phosphosites was set as *p* value < 0.05.

e. Pairwise comparison of each phosphosite between control and virus-infected groups was performed with Student’s *t* test. The *p* value and fold change of each quantified site in the liver was plotted. The cutoff of differentially expressed phosphosites was set as *p* value < 0.05.

f. Differentially expressed phosphosites in the lung and liver. The left panel shows the counts of differentially expressed sites in the lung and liver. We divided mean values of virus-infected group by mean values of control group to get fold change (FC). Up is set as *p* value < 0.05 and FC > 1 while down is set as *p* value < 0.05 and FC < 1. The right panel shows the intersection of differentially expressed phosphosites between each dataset. Compared to the control, 1,162 sites (Up: 541; Down: 621; see also supplementary TableS10) in 873 proteins in the lung and 960 sites (Up: 573; Down: 387; see also supplementary TableS11) in 712 proteins in the liver were differentially expressed in the infected group.


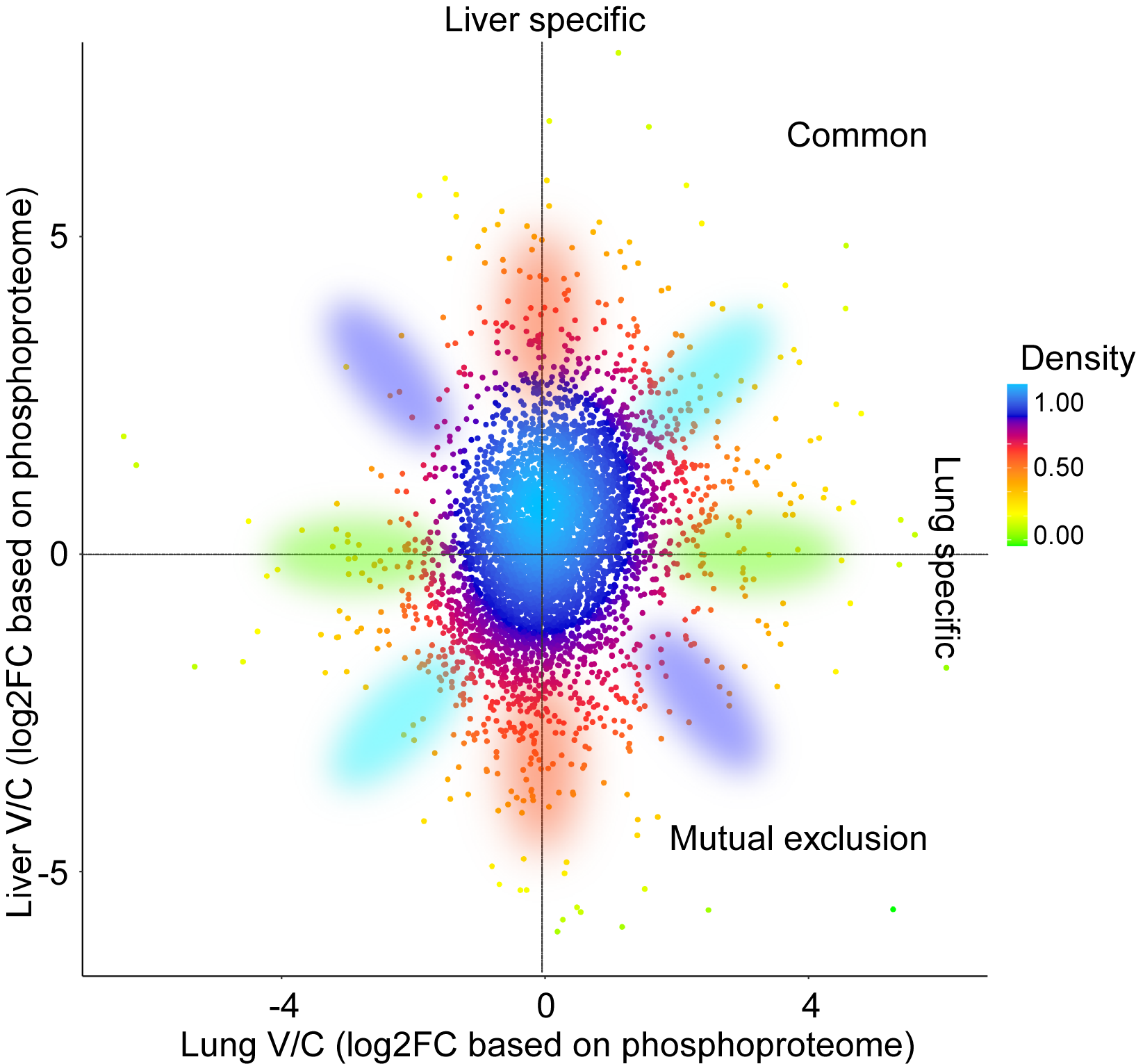


**Supplementary Figure. S6** Comparison between the shared phosphosites in the lung and liver of rhesus macaques after SARA-CoV-2 infection.

The intersection of quantifiable phosphosites in the lung and liver were extracted and their fold changes in these two organs after infection were used to construct the scatter plot. The green marked parts represent phosphosites that were differentially expressed only in the lung (lung specific). The orange marked parts represents phosphosites that were differentially expressed only in the liver (liver specific). The blue and purple marked parts represent phosphosites that were differentially expressed both in the lung and liver. Blue marks mean the fold changes of phosphosites have coincident trends in both the lung and liver after SARS-CoV-2 infection (common). Purple marks mean the fold changes of phosphosites have opposite trends in the lung and liver after infection (mutual exclusion).


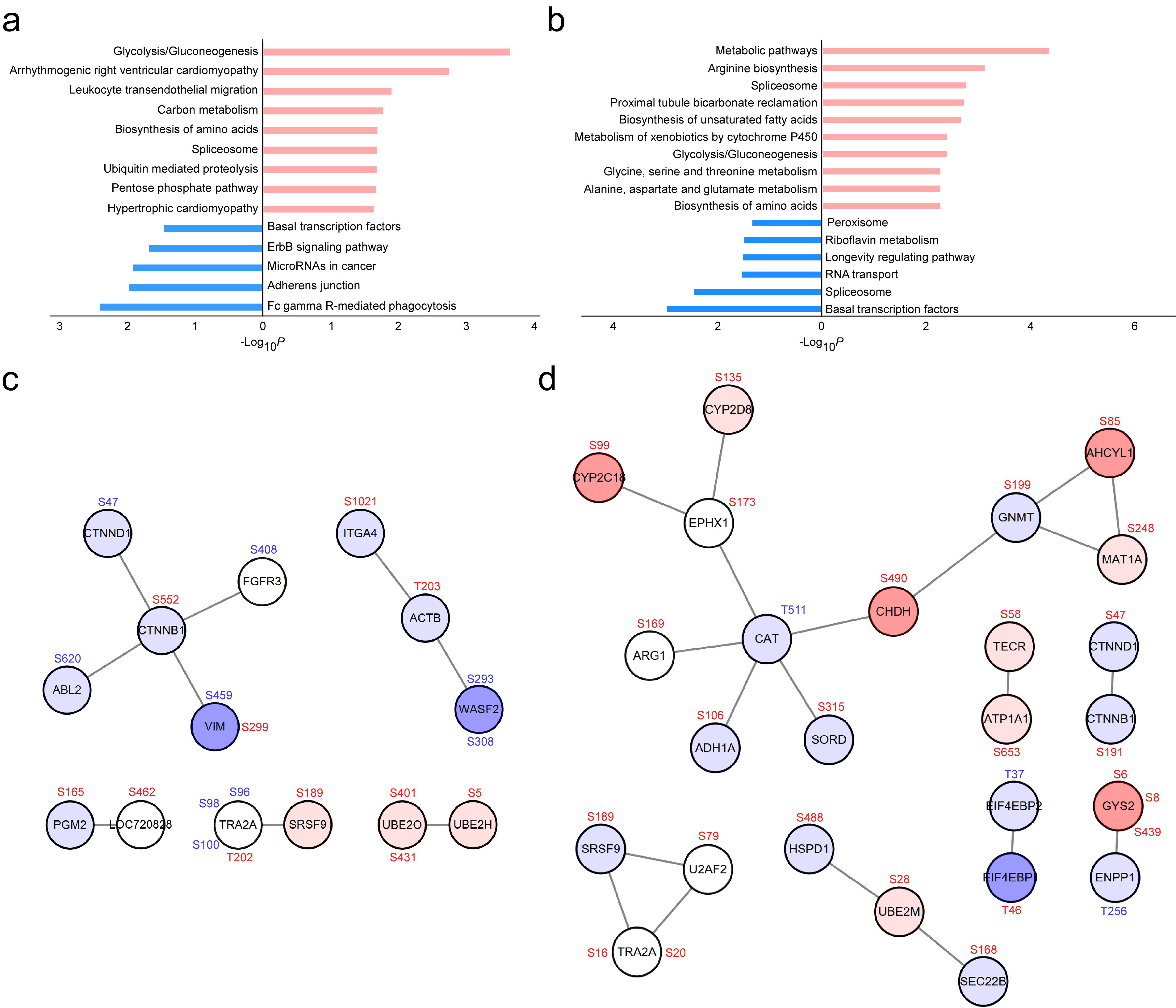


**Supplementary Figure. S7** KEGG and PPI analysis of proteins containing differentially expressed phosphosites in the lung and liver of rhesus macaques.

a. KEGG enrichment analysis of proteins containing differentially expressed phosphosites in the lung of control and virus-infected rhesus macaques. Red: proteins with upregulated phosphosites. Blue: proteins with downregulated phosphosites. See also supplementary TableS12.

b. KEGG enrichment analysis of proteins containing differentially expressed phosphosites in the liver of control and virus-infected rhesus macaques. Red: proteins with upregulated phosphosites. Blue: proteins with downregulated phosphosites. See also supplementary TableS13.

c-d. PPI networks of proteins which contained differentially expressed phosphosites and appeared in enriched KEGG pathways in the phosphoproteome. Blue fill: the protein was downregulated in the proteome (light blue: Student’s *t* test *p* value > 0.05; dark: *p* value < 0.05). Red fill: the protein was downregulated in the proteome (pink: Student’s *t* test *p* value > 0.05; dark: *p* value < 0.05). White fill: the protein had no quantification information in the proteome. Red characters: the phosphosite was upregulated in the phosphoproteome. Blue characters: the phosphosites was downregulated in the phosphoproteome. Figure c presents the PPI network in the lung. Figure d presents the PPI network in the liver.


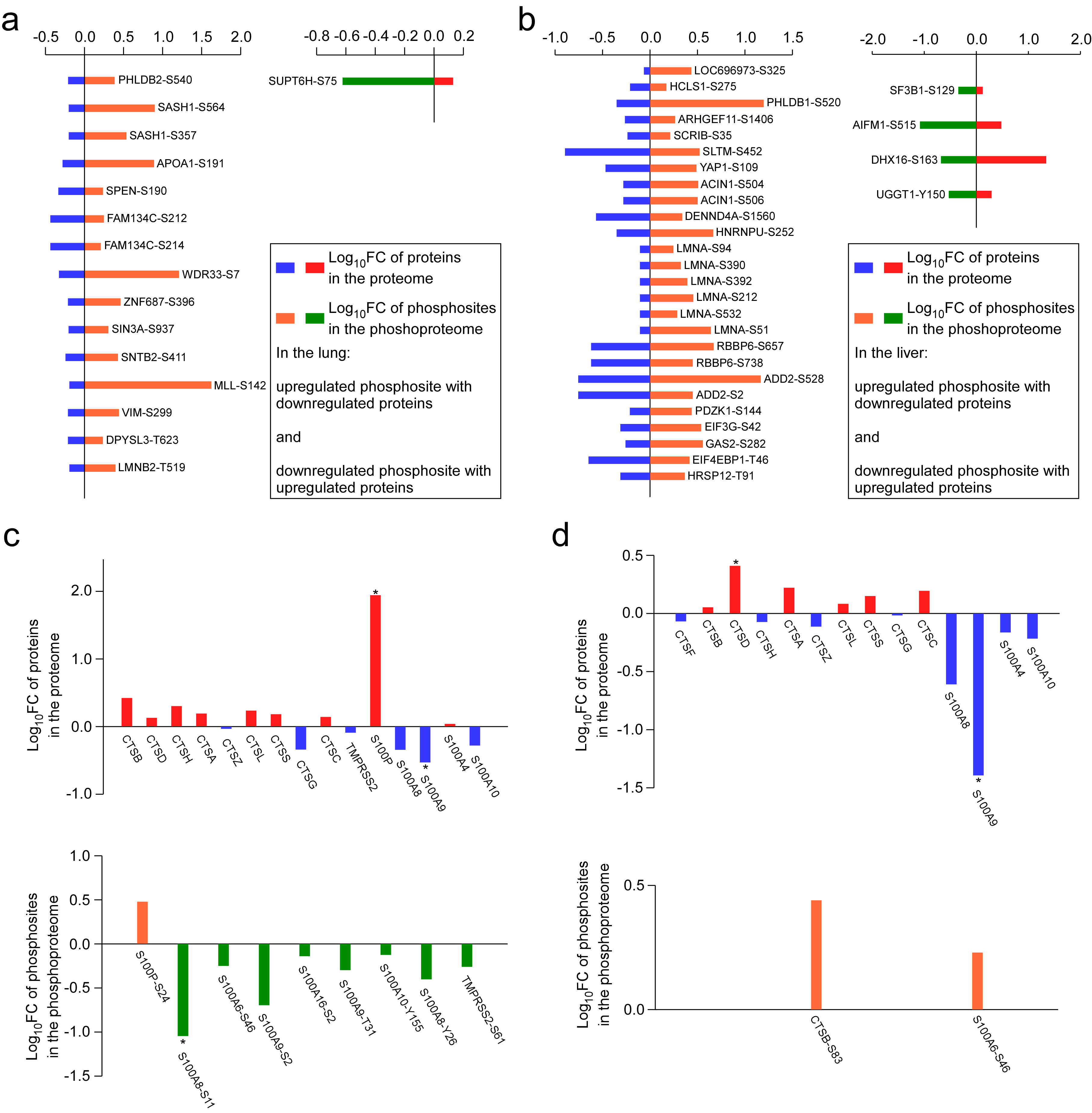


**Supplementary Figure. S8** Alteration of specific molecules in the lung and liver of rhesus macaques.

a-b. Differentially expressed protein/phosphosite pairs which had opposite FC in the proteome and phosphoproteome. Red: log_10_FC of the protein in the proteome was over 0. Blue: log_10_FC of the protein in the proteome was less than 0. Orange: log_10_FC of the phosphosite in the phosphoproteome was over 0. Green: log_10_FC of the phosphosite in the phosphoproteome was less than 0. Figure a presents the protein/phosphosite pairs in the lung. Figure b presents the protein/phophosite pairs in the liver.

c-d. Alterations of Cathepsins, TMPRSS2, and S100 calcium binding proteins, which were reported to play important roles in the entrance of SARS-CoV-2 into cells,^9,10^ in the proteome and phosphoproteome. ACE2 was detected in neither omics. Red: log_10_FC of the protein in the proteome was over 0. Blue: log_10_FC of the protein in the proteome was less than 0. Orange: log_10_FC of the phosphosite in the phosphoproteome was over 0. Green: log_10_FC of the phosphosite in the phosphoproteome was less than 0 (*, *p* value of Student’s *t* test was less than 0.05). Figure c presents the proteins/phosphosites in the lung. Figure d presents the proteins/phophosites in the liver.


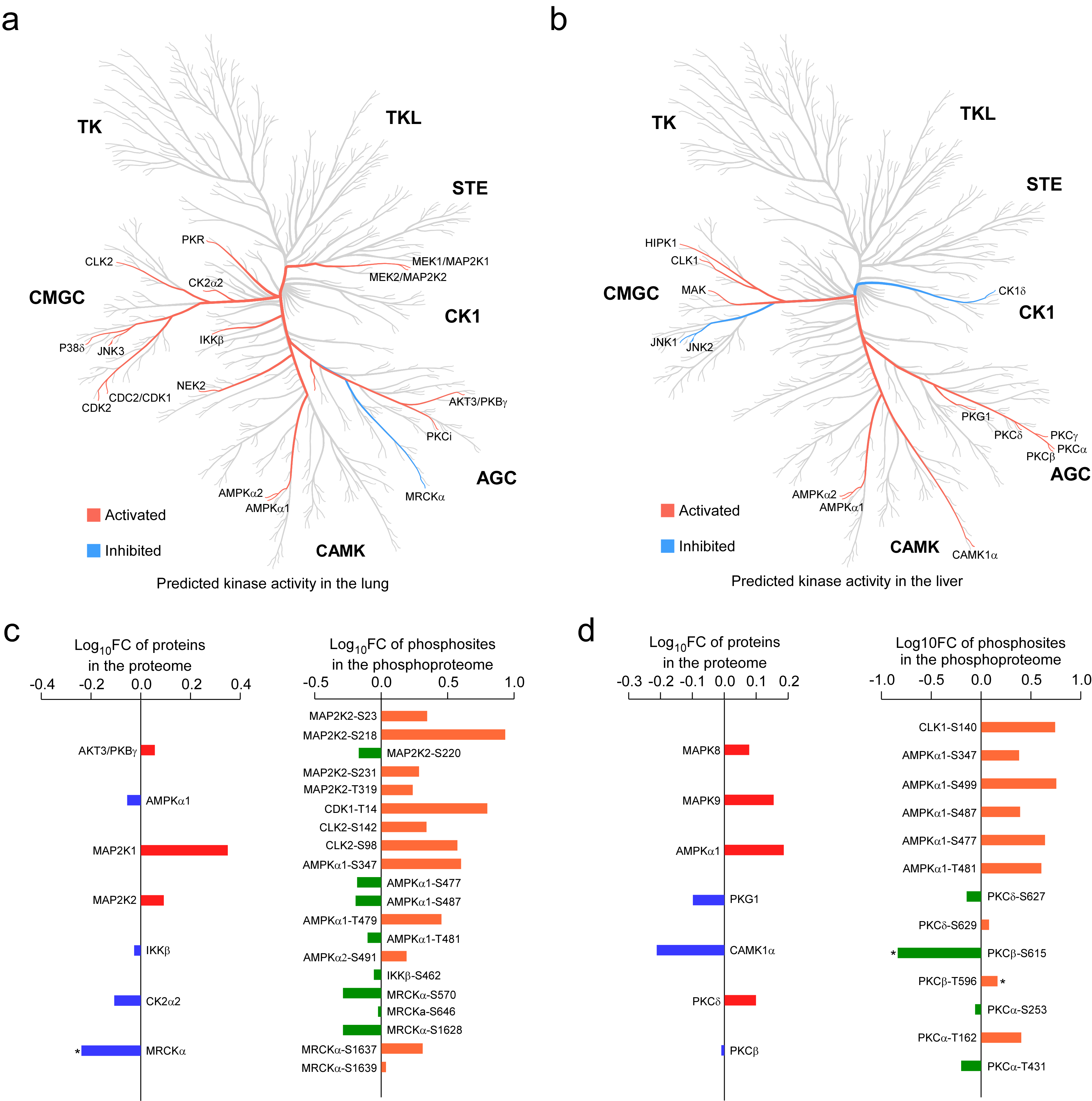


**Supplementary Figure. S9** Kinase analyses in the lung and liver of rhesus macaques.

a. Prediction of kinase activity using NetworKIN, which is based on identified phosphosites in the lung. Red: activated kinases after SARS-CoV-2 infection. Blue: inhibited kinases after infection. See also supplementary TableS14.

b. Prediction of kinase activity using NetworKIN, which is based on identified phosphosites in the liver. Red: activated kinases after SARS-CoV-2 infection. Blue: inhibited kinases after infection. See also supplementary TableS15.

c-d. Predicted kinases were looked back in the proteome and phosphoproteome. Matched quantifiable kinases and their phosphosites in the lung (c) and liver (d) were presented in the figure. Red: log_10_FC of the protein in the proteome was over 0. Blue: log_10_FC of the protein in the proteome was less than 0. Orange: log_10_FC of the phosphosite in the phosphoproteome was over 0. Green: log_10_FC of the phosphosite in the phosphoproteome was less than 0 (*, *p* value of Student’s *t* test was less than 0.05).


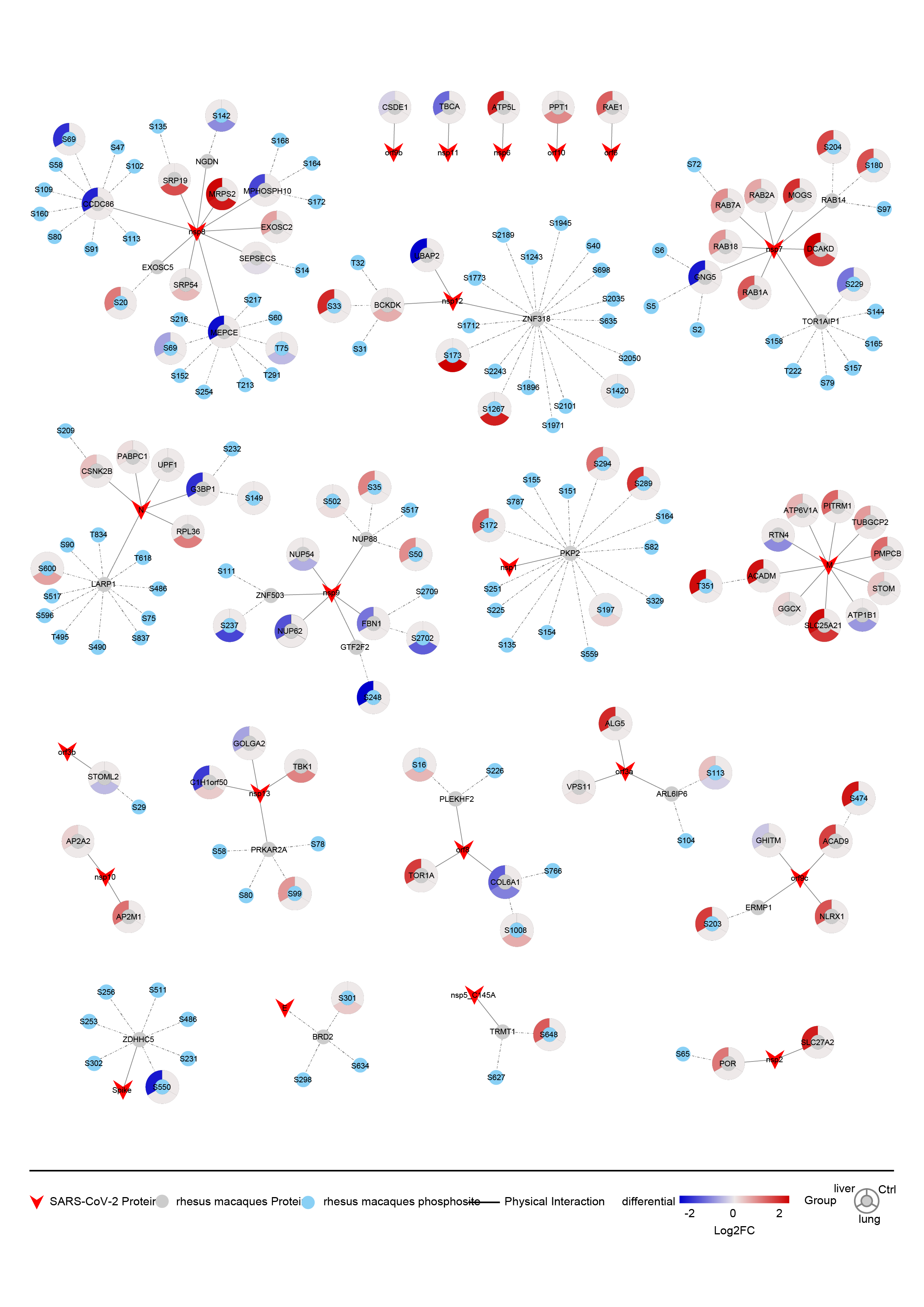


**Supplementary Figure. S10** Interactions among SARS-CoV-2 viral proteins and differentially expressed host proteins and phosphosites.

According to the 332 high-confidence protein-protein interactions between SARS-CoV-2 and human proteins constructed by Gordon, *et al.* using affinity-purification mass spectrometry,^11^ we matched proteins of rhesus macaque to human being by blast and constructed a network among viral proteins and differentially expressed host proteins and phosphosites in this study. The red polygons represent SARS-CoV-2 viral proteins. The grey circles represent proteins in rhesus macaques. The blue circles represent phosphosites in rhesus macaques. The peripheral colored circles around grey or blue circles mean that the protein/phosphosite was differentially expressed in the lung or liver; the colors in the peripheral circles represent the abundance of proteins/phosphosites in the lung and liver. No peripheral colored circle means the protein/phosphosite is identified or differentially expressed in neither lung nor liver of rhesus macaques. Also see supplementary TableS25.

**Supplementary tables**

**Supplementary Table S1**. Animal information in this study.

**Supplementary Table S2**. All the quantifiable proteins in the lung of rhesus macaques.

**Supplementary Table S3**. All the quantifiable proteins in the liver of rhesus macaques.

**Supplementary Table S4**. Comparison between the intersected proteins in the lung and liver of rhesus macaques.

**Supplementary Table S5**. Differentially expressed proteins between COVID-19 and control lung samples of rhesus macaques.

**Supplementary Table S6**. Differentially expressed proteins between COVID-19 and control liver samples of rhesus macaques.

**Supplementary Table S7**. Comparison between differentially expressed proteins in the lung and liver of rhesus macaques

**Supplementary Table S8**. KEGG pathways enriched for differentially expressed proteins in the lung of rhesus macaques.

**Supplementary Table S9**. KEGG pathways enriched for differentially expressed proteins in the liver of rhesus macaques.

**Supplementary Table S10**. All the quantifiable phosphosites in the lung of rhesus macaques.

**Supplementary Table S11**. All the quantifiable phosphosites in the liver of rhesus macaques.

**Supplementary Table S12**. Comparison between the intersected phosphosites in the lung and liver of rhesus macaques

**Supplementary Table S13**. Differentially expressed phosphosites between COVID-19 and control lung samples of rhesus macaques.

**Supplementary Table S14**. Differentially expressed phosphosites between COVID-19 and control liver samples of rhesus macaques.

**Supplementary Table S15**. Comparison between differentially expressed phosphosites in the lung and liver of rhesus macaques

**Supplementary Table S16**. KEGG pathways enriched for proteins containing differentially expressed phosphosites in the lung of rhesus macaques.

**Supplementary Table S17**. KEGG pathways enriched for proteins containing differentially expressed phosphosites in the liver of rhesus macaques.

**Supplementary Table S18**. Predicted kinase activity in the lung of rhesus macaques.

**Supplementary Table S19**. Predicted kinase activity in the liver of rhesus macaques.

**Supplementary Table S20**. DrugBank-based FDA-approved drugs for predicted kinases in this study.

**Supplementary Table S21**. Comparison of differentially expressed proteins between this research and Nie's report in the lung.

**Supplementary Table S22**. Comparison of differentially expressed proteins between rhesus macaques and Nie's report in the liver.

**Supplementary Table S23**. Comparison of differentially expressed phosphosites between rhesus macaques and Bouhaddou's report in the lung.

**Supplementary Table S24**. Comparison of differentially expressed phosphosites between rhesus macaques and Bouhaddou's report in the liver.

**Supplementary Table S25**. Prey proteins in the rhesus macaques based on previous interactome.

**References**

1 Cox, J. & Mann, M. MaxQuant enables high peptide identification rates, individualized p.p.b.-range mass accuracies and proteome-wide protein quantification. *Nat Biotechnol*. **26**, 1367-1372, (2008).

2 Tyanova, S. *et al.* The Perseus computational platform for comprehensive analysis of (prote)omics data. *Nat Methods*. **13**, 731-740, (2016).

3 Xie, C. *et al.* KOBAS 2.0: a web server for annotation and identification of enriched pathways and diseases. *Nucleic Acids Res*. **39**, W316-322, (2011).

4 Kanehisa, M. *et al.* KEGG for linking genomes to life and the environment. *Nucleic Acids Res*. **36**, D480-484, (2008).

5 Linding, R. *et al.* NetworKIN: a resource for exploring cellular phosphorylation networks. *Nucleic Acids Res*. **36**, D695-699, (2008).

6 Horn, H. *et al.* KinomeXplorer: an integrated platform for kinome biology studies. *Nat Methods*. **11**, 603-604, (2014).

7 Subramanian, A. *et al.* Gene set enrichment analysis: a knowledge-based approach for interpreting genome-wide expression profiles. *Proc Natl Acad Sci U S A*. **102**, 15545-15550, (2005).

8 Chartier, M., Chenard, T., Barker, J. & Najmanovich, R. Kinome Render: a stand-alone and web-accessible tool to annotate the human protein kinome tree. *PeerJ*. **1**, e126, (2013).

9 Wu, M. *et al.* Transcriptional and proteomic insights into the host response in fatal COVID-19 cases. *Proc Natl Acad Sci U S A*. **117**, 28336-28343, (2020).

10 Nie, X. *et al.* Multi-organ proteomic landscape of COVID-19 autopsies. *Cell*. **184**, 775-791 e714, (2021).

11 Gordon, D. E. *et al.* A SARS-CoV-2 protein interaction map reveals targets for drug repurposing. *Nature*. **583**, 459-468, (2020).
